# Supplementary material for: Performance of Localized-Orbital Coupled-Cluster Approaches for the Conformational Energies of Longer n-Alkane Chains
Source: J Phys Chem A. 2022 Dec 12;126(50):9375–91. doi: 10.1021/acs.jpca.2c06407 (PMC9791657; doi:10.1021/acs.jpca.2c06407)
Supplement: Supplementary file 1 — jp2c06407_si_001.pdf [file jp2c06407_si_001.pdf]

Supporting Information for:

Performance of Localized-Orbital Coupled Cluster Approaches for the Conformational Energies of Longer n-alkane Chains

Golokesh Santra* and Jan M.L. Martin*

Department of Molecular Chemistry and Materials Science, Weizmann Institute of Science,

7610001 Reḥovot, Israel.

Email: gershom@weizmann.ac.il

golokesh.santra@weizmann.ac.il

Table S1: Performance of RI-MP2 and RI-MP2-F12 methods with different basis set size for the conformer energies of *n*-dodecane (C_nH_{2n+2}, n=12). All the results are in kcal/mol unit.

# Conf.	New Ref.[a]	RI-MP2-F12				RI-MP2							
		VDZ-F12	VTZ-F12	VQZ-F12	V{T,Q}Z-F12	def2-TZVPP	def2-QZVPP	def2-{T,Q}ZVPP	AVTZ	AVQZ	AV5Z	AV{T,Q}Z	AV{Q,5}Z
1	1.82	1.62	1.61	1.61	1.60	1.48	1.56	1.62	1.42	1.56	1.59	1.69	1.62
2	2.05	1.63	1.62	1.61	1.60	1.29	1.52	1.73	1.22	1.49	1.56	1.75	1.64
3	2.49	2.17	2.16	2.16	2.15	1.85	2.06	2.26	1.82	2.06	2.11	2.28	2.18
4	3.16	2.70	2.71	2.71	2.70	2.23	2.56	2.86	1.95	2.45	2.61	2.92	2.79
5	3.66	3.49	3.50	3.50	3.49	3.25	3.42	3.59	3.15	3.39	3.46	3.61	3.53
6	3.88	3.58	3.58	3.59	3.58	3.23	3.47	3.70	3.08	3.41	3.52	3.72	3.65
7	4.16	3.89	3.89	3.89	3.88	3.56	3.79	4.00	3.47	3.76	3.83	4.03	3.92
8	4.31	3.90	3.90	3.90	3.89	3.44	3.75	4.03	3.16	3.62	3.79	4.06	3.99
9	4.89	4.59	4.60	4.60	4.59	4.23	4.48	4.72	4.13	4.45	4.54	4.76	4.63
10	5.45	5.11	5.12	5.13	5.11	4.65	4.98	5.29	4.41	4.88	5.03	5.32	5.20
11	5.99	5.63	5.64	5.64	5.63	5.23	5.51	5.78	4.98	5.40	5.54	5.79	5.72
12	6.56	6.28	6.28	6.28	6.27	5.97	6.19	6.39	5.83	6.12	6.22	6.40	6.33
**MAD (kcal/mol)**	**ref**	**0.32**	**0.32**	**0.32**	**0.33**	**0.67**	**0.43**	**0.20**	**0.82**	**0.49**	**0.39**	**0.18**	**0.27**
**RMSD (kcal/mol)**		**0.33**	**0.33**	**0.33**	**0.34**	**0.69**	**0.44**	**0.21**	**0.85**	**0.50**	**0.40**	**0.19**	**0.28**
**MSD (kcal/mol)**		**-0.32**	**-0.32**	**-0.32**	**-0.33**	**-0.67**	**-0.43**	**-0.20**	**-0.82**	**-0.49**	**-0.39**	**-0.18**	**-0.27**
**MAD (kcal/mol)**	**0.33**	**0.01**	**0.01**	**0.01**	**ref**	**0.34**	**0.10**	**0.13**	**0.49**	**0.16**	**0.06**	**0.15**	**0.06**
**RMSD (kcal/mol)**	**0.34**	**0.01**	**0.01**	**0.01**		**0.35**	**0.10**	**0.13**	**0.52**	**0.17**	**0.06**	**0.16**	**0.07**
**MSD (kcal/mol)**	**0.33**	**0.01**	**0.01**	**0.01**		**-0.34**	**-0.10**	**0.13**	**-0.49**	**-0.16**	**-0.06**	**0.15**	**0.06**

[a]MP2-F12/V{T,Q}Z-F12 + [CCSD(F12*)-MP2-F12]/cc-pVTZ-F12 + [DF-CCSD(T)-DF-CCSD]/AV{D,T}Z

Table S2: Performance of six HLCs relative to canonical [DF-CCSD(T) - DF-MP2] with AVTZ throughout. Total [CCSD(T)-MP2] contributions has been divided into [CCSD-MP2] and (T) parts for better understanding of error compensation in different localized orbital methods. All the results are in kcal/mol unit.

	# Conf. C_nH_{2n+2} n=12	[DF-CCSD(T) - DF-MP2]	HLC6	HLC7	HLC8	HLC3	HLC15	HLC16	[DLPNO-CCSD(T_1) - LMP2]/TightPNO
For [CCSD-MP2]	**1**	0.48	0.46	0.46	0.46	0.50	0.45	0.48	0.52
	**2**	0.97	0.94	0.93	0.92	0.99	0.89	0.93	1.01
	**3**	0.81	0.77	0.77	0.76	0.83	0.75	0.79	0.89
	**4**	1.28	1.24	1.21	1.21	1.30	1.15	1.20	1.20
	**5**	0.58	0.56	0.55	0.55	0.60	0.53	0.56	0.60
	**6**	0.91	0.88	0.86	0.85	0.92	0.82	0.86	0.92
	**7**	0.79	0.75	0.75	0.75	0.81	0.72	0.76	0.82
	**8**	1.26	1.23	1.19	1.19	1.29	1.15	1.18	1.20
	**9**	0.93	0.90	0.88	0.87	0.95	0.84	0.89	0.95
	**10**	1.15	1.11	1.08	1.08	1.17	1.03	1.08	1.12
	**11**	1.17	1.14	1.11	1.10	1.20	1.05	1.09	1.16
	**12**	0.93	0.89	0.88	0.88	0.96	0.85	0.89	0.97
	**MAD**		**0.034**	**0.050**	**0.053**	**0.021**	**0.087**	**0.047**	**0.037**
	**RMSD**	**REF**	**0.035**	**0.052**	**0.055**	**0.022**	**0.091**	**0.053**	**0.043**
	**MSD**		**-0.034**	**-0.050**	**-0.053**	**0.021**	**-0.087**	**-0.047**	**0.007**
Just For (T)	**1**	-0.25	-0.26	-0.23	-0.24	-0.24	-0.21	-0.23	-0.26
	**2**	-0.49	-0.52	-0.46	-0.47	-0.48	-0.44	-0.45	-0.49
	**3**	-0.44	-0.44	-0.41	-0.41	-0.43	-0.39	-0.40	-0.44
	**4**	-0.75	-0.82	-0.71	-0.70	-0.73	-0.67	-0.69	-0.70
	**5**	-0.39	-0.42	-0.38	-0.38	-0.38	-0.35	-0.36	-0.39
	**6**	-0.57	-0.61	-0.54	-0.54	-0.56	-0.51	-0.52	-0.55
	**7**	-0.49	-0.51	-0.47	-0.46	-0.48	-0.43	-0.44	-0.48
	**8**	-0.78	-0.84	-0.73	-0.72	-0.75	-0.69	-0.71	-0.72
	**9**	-0.60	-0.63	-0.57	-0.57	-0.59	-0.53	-0.55	-0.58
	**10**	-0.77	-0.81	-0.72	-0.72	-0.74	-0.68	-0.70	-0.73
	**11**	-0.76	-0.82	-0.72	-0.72	-0.74	-0.67	-0.69	-0.71
	**12**	-0.62	-0.64	-0.60	-0.60	-0.61	-0.55	-0.57	-0.60
	**MAD**		**0.034**	**0.031**	**0.033**	**0.017**	**0.067**	**0.051**	**0.025**
	**RMSD**	**REF**	**0.039**	**0.033**	**0.036**	**0.019**	**0.070**	**0.053**	**0.032**
	**MSD**		**-0.034**	**0.031**	**0.033**	**0.017**	**0.067**	**0.051**	**0.022**
For total [CCSD(T)-MP2]	**1**	0.24	0.20	0.23	0.22	0.25	0.23	0.25	0.26
	**2**	0.48	0.42	0.46	0.46	0.50	0.46	0.48	0.52
	**3**	0.37	0.33	0.36	0.35	0.40	0.36	0.39	0.45
	**4**	0.52	0.42	0.50	0.51	0.58	0.49	0.51	0.51
	**5**	0.19	0.13	0.17	0.17	0.22	0.18	0.20	0.21
	**6**	0.33	0.27	0.31	0.32	0.37	0.32	0.34	0.38
	**7**	0.31	0.24	0.28	0.28	0.34	0.29	0.32	0.33
	**8**	0.48	0.39	0.46	0.47	0.54	0.46	0.47	0.48
	**9**	0.33	0.27	0.31	0.31	0.37	0.31	0.34	0.37
	**10**	0.38	0.30	0.36	0.36	0.43	0.35	0.38	0.39
	**11**	0.40	0.31	0.38	0.38	0.46	0.38	0.40	0.44
	**12**	0.31	0.25	0.28	0.28	0.35	0.30	0.32	0.37
	**MAD**		**0.068**	**0.019**	**0.020**	**0.038**	**0.020**	**0.009**	**0.033**
	**RMSD**	**REF**	**0.070**	**0.020**	**0.021**	**0.040**	**0.022**	**0.010**	**0.038**
	**MSD**		**-0.068**	**-0.019**	**-0.020**	**0.038**	**-0.020**	**0.003**	**0.029**

Table S3: Performance of localized orbital based high-level corrections relative to the canonical [CCSD(F12*) − MP2-F12]/VTZ-F12 + (T)/AV{D,T}Z correction used for the reference conformer energies of *n*-dodecane.

	# Conformers in the ACONF12 set												MAD (kcal/mol)	RMSD (kcal/mol)	MSD (kcal/mol)
	**01**	**02**	**03**	**04**	**05**	**06**	**07**	**08**	**09**	**10**	**11**	**12**			
	0.22	0.45	0.34	0.46	0.17	0.30	0.28	0.42	0.30	0.34	0.36	0.29		**Ref.**	
**HLC1**[a]	0.24	0.48	0.37	0.52	0.19	0.33	0.31	0.48	0.33	0.38	0.40	0.31	0.035	0.038	0.035
**HLC2**[b]	0.25	0.51	0.43	0.50	0.20	0.36	0.32	0.47	0.35	0.37	0.44	0.35	0.050	0.053	0.050
**HLC3**[c]	0.25	0.50	0.40	0.58	0.22	0.37	0.34	0.54	0.37	0.43	0.46	0.35	0.073	0.078	0.073
**HLC4**[d]	0.27	0.55	0.43	0.63	0.25	0.42	0.38	0.61	0.43	0.51	0.53	0.42	0.125	0.132	0.125
**HLC5**[e]	0.33	0.58	0.46	0.58	0.24	0.41	0.43	0.58	0.43	0.47	0.53	0.44	0.130	0.132	0.130
**HLC6**[f]	0.20	0.42	0.33	0.42	0.13	0.27	0.24	0.39	0.27	0.30	0.31	0.25	0.032	0.034	-0.032
**HLC7**[g]	0.23	0.46	0.36	0.50	0.17	0.31	0.28	0.46	0.31	0.36	0.38	0.28	0.017	0.021	0.016
**HLC8**[h]	0.22	0.46	0.35	0.51	0.17	0.32	0.28	0.47	0.31	0.36	0.38	0.28	0.016	0.023	0.015
**HLC9**[i]	0.21	0.44	0.35	0.45	0.15	0.29	0.26	0.43	0.30	0.34	0.35	0.28	0.009	0.011	-0.007
**HLC10**[j]	0.26	0.50	0.40	0.55	0.20	0.36	0.32	0.51	0.36	0.42	0.44	0.33	0.059	0.062	0.059
**HLC11**[k]	0.24	0.48	0.38	0.51	0.19	0.33	0.30	0.47	0.33	0.38	0.40	0.31	0.031	0.032	0.031
**HLC12**[l]	0.27	0.52	0.42	0.58	0.23	0.39	0.35	0.55	0.39	0.45	0.47	0.37	0.088	0.091	0.088
**HLC13**[m]	0.22	0.46	0.36	0.47	0.16	0.31	0.27	0.45	0.31	0.36	0.38	0.30	0.012	0.014	0.010
**HLC14**[n]	**0.22**	**0.45**	**0.36**	**0.46**	**0.17**	**0.29**	**0.28**	**0.42**	**0.31**	**0.34**	**0.36**	**0.28**	**0.006**	**0.008**	**0.000**
**HLC15**[o]	0.23	0.46	0.36	0.49	0.18	0.32	0.29	0.46	0.31	0.35	0.38	0.30	0.015	0.018	0.015
**HLC16**[p]	0.25	0.48	0.39	0.51	0.20	0.34	0.32	0.47	0.34	0.38	0.40	0.32	0.038	0.039	0.038
**HLC17**[q]	0.25	0.47	0.38	0.50	0.20	0.33	0.31	0.47	0.34	0.38	0.40	0.33	0.035	0.035	0.035
**HLC18**[r]	0.25	0.48	0.39	0.52	0.22	0.35	0.33	0.49	0.36	0.40	0.42	0.35	0.052	0.053	0.052
**HLC19**[s]	0.26	0.48	0.39	0.51	0.21	0.34	0.33	0.48	0.35	0.39	0.42	0.35	0.048	0.048	0.048
**HLC20**[t]	0.26	0.49	0.40	0.53	0.23	0.36	0.34	0.50	0.37	0.41	0.44	0.36	0.062	0.063	0.062
**HLC21**[u]	0.28	0.51	0.41	0.56	0.24	0.38	0.36	0.53	0.40	0.44	0.47	0.40	0.087	0.089	0.087
**HLC22**[v]	0.29	0.52	0.43	0.56	0.25	0.39	0.37	0.52	0.40	0.44	0.46	0.39	0.091	0.091	0.091

[a][DF-CCSD(T) − RI-MP2]/AVTZ
[b][DLPNO-CCSD(T1) − LMP2]/TightPNO/CPS{6,7}/AVTZ
[c][DLPNO-CCSD(T1) − LMP2]/VeryTightPNO/AVTZ
[d][DLPNO-CCSD(T1) − LMP2]/ VeryTightPNO/AV{T,Q}Z
[e][DLPNO-CCSD(T1) − LMP2]/TightPNO/CPS{6,7}/AV{T,Q}Z
[f][LNO-CCSD(T) − LMP2]/Tight/AVTZ
[g][LNO-CCSD(T) − LMP2]/vTight/AVTZ
[h][LNO-CCSD(T) − LMP2]/vvTight/AVTZ
[i][LNO-CCSD(T) − LMP2]/Tight/AVQZ
[j][LNO-CCSD(T) − LMP2]/vTight/AVQZ
[k][LNO-CCSD(T) − LMP2]/vTight/AV5Z
[l][LNO-CCSD(T) − LMP2]/vTight/AV{T,Q}Z
[m][LNO-CCSD(T) − LMP2]/Tight/AV{T,Q}Z
[n][LNO-CCSD(T) − LMP2]/vTight/AV{Q,5}Z
[o][PNO-LCCSD(T) − LMP2]/Default/AVTZ
[p][PNO-LCCSD(T) − LMP2]/Tight/AVTZ
[q][PNO-CCSD(T) − LMP2]/Default/AVQZ
[r][PNO-CCSD(T) − LMP2]/Tight/AVQZ
[s][PNO-LCCSD(T) − LMP2]/Default/AV{T,Q}Z
[t][PNO-LCCSD(T) − LMP2]/Tight/AV{T,Q}Z
[u][PNO-LCCSD(Ts)-F12b − LMP2-F12]/ Default/VTZ-F12
[v][PNO-LCCSD(Ts)-F12b − LMP2-F12]/Tight/VTZ-F12

Table S4: Conformer energies of *n*-dodecane, *n*-hexadecane, and *n*-icosane with three high-level corrections (HLC14, HLC8, and HLC13) using localized orbital methods. For convenience, we have retained the numbering and ordering of different conformers from ref.[1] ; conformer energies of *n*-dodecane and *n*-hexadecane are relative to the all-trans conformer **0**, while those for *n*-icosane are relative to the "hairpin" conformer **00**.

# Conf. C_nH_{2n+2} n=12	RI-MP2-F12/V{T,Q}Z-F12 + HLC			# Conf. C_nH_{2n+2} n=16	RI-MP2-F12/V{T,Q}Z-F12 + HLC			# Conf. C_nH_{2n+2} n=20	RI-MP2-F12/V{T,Q}Z-F12 + HLC		
	HLC14[a]	HLC8[b]	HLC13[c]		HLC14[a]	HLC8[b]	HLC13[c]		HLC14[a]	HLC8[b]	HLC13[c]
1	1.82	1.82	1.79	00	-0.49	-0.39	-0.49	0	2.20	2.06	2.19
2	2.06	2.06	2.01	1	2.15	2.19	2.10	1	4.15	4.10	4.07
3	2.51	2.50	2.47	3	2.54	2.57	2.47	5	4.81	4.67	4.75
4	3.16	3.21	3.10	4	2.68	2.68	2.63	6	4.99	4.87	4.91
5	3.65	3.66	3.61	2	2.94	2.98	2.87	7	5.27	5.14	5.21
6	3.87	3.89	3.83	6	3.24	3.23	3.14	3	4.85	4.81	4.74
7	4.16	4.16	4.11	7	3.28	3.29	3.21	11	5.60	5.47	5.52
8	4.31	4.36	4.25	5	3.34	3.35	3.25	10	5.58	5.46	5.50
9	4.90	4.90	4.84	8	3.68	3.68	3.62	4	5.31	5.26	5.23
10	5.45	5.48	5.40	9	3.98	3.99	3.93	12	5.74	5.61	5.69
11	5.99	6.01	5.92	10	4.08	4.12	4.03	8	5.28	5.27	5.21
12	6.55	6.56	6.50	11	4.35	4.36	4.31	2	5.05	5.11	4.95
				12	4.54	4.54	4.47	9	5.77	5.65	5.67
				14	4.95	4.95	4.89	16	6.01	5.89	5.91
				13	5.02	5.00	4.95	13	6.12	5.99	6.03
				15	5.84	5.94	5.71	17	6.33	6.24	6.24
				16	6.11	6.15	6.02	15	6.52	6.39	6.41
								19	6.65	6.52	6.56
								14	6.38	6.36	6.28
								18	6.74	6.73	6.63
								20	7.94	7.97	7.81

[a] HLC14 = [LNO-CCSD(T) − LMP2]/vTight/AV{Q,5}Z
[b] HLC8 = [LNO-CCSD(T) − LMP2]/vvTight/AVTZ
[c] HLC13 = [LNO-CCSD(T) − LMP2]/Tight/AV{T,Q}Z

Table S5: ACONF16 conformer energies relative to the **00** (hairpin) conformer.

# Conf. ACONF16	MP2-F12/V{T,Q}Z-F12+HLC		
	HLC14[(a)]	HLC8[(b)]	HLC13[(c)]
0	0.49	0.39	0.49
1	2.64	2.58	2.58
3	3.02	2.96	2.96
4	3.16	3.07	3.12
2	3.43	3.38	3.36
6	3.72	3.62	3.63
7	3.77	3.69	3.69
5	3.83	3.75	3.73
8	4.17	4.08	4.11
9	4.47	4.39	4.41
10	4.57	4.51	4.52
11	4.84	4.75	4.79
12	5.03	4.94	4.96
14	5.43	5.34	5.38
13	5.51	5.40	5.44
15	6.33	6.33	6.19
16	6.59	6.55	6.51

[(a)] HLC14 = [LNO-CCSD(T) − LMP2]/vTight/AV{Q,5}Z
[(b)] HLC8 = [LNO-CCSD(T) − LMP2]/vvTight/AVTZ
[(c)] HLC13 = [LNO-CCSD(T) − LMP2]/Tight/AV{T,Q}Z

Table S6: Performance of the composite LNO-CCSD(T), PNO-LCCSD(T), DLPNO-CCSD(T_0), and DLPNO-CCSD(T_1) methods with respect to the revised ACONFL reference data. Heatmapping is from red (worst) via yellow to green (best).[a]

Method Details	Composite methods	c_1	MAD (kcal/mol)				MSD	RMSD
			ACONFL	ACONF12	ACONF16	ACONF20	(kcal/mol)	(kcal/mol)
Coefficients (c_1) are taken from the "Raw" category of Ref.[2]								
LNO-CCSD(T)	Normal {T,Q} + c_1[vTight – Normal]/T	0.93	0.23	0.26	0.28	0.18	0.09	0.26
	Normal {Q,5} + c_1[vTight – Normal]/T	1.09	0.18	0.15	0.28	0.12	0.17	0.21
	Tight {T,Q} + c_1[vTight – Tight]/T	0.79	0.23	0.21	0.25	0.22	0.05	0.25
	Tight {T,Q} + c_1[vvTight – Tight]/T	0.72	0.23	0.21	0.24	0.23	0.04	0.25
	Tight {Q,5} + c_1[vTight – Tight]/T	1.26	0.12	0.11	0.13	0.12	0.03	0.13
	Tight{Q,5} + c_1[vTight – Tight]/Q	1.14	0.08	0.05	0.08	0.09	0.00	0.09
	Tight {Q,5} + c_1[vvTight – Tight]/T	1.11	0.12	0.11	0.11	0.13	0.02	0.13
	vTight {T,Q} + c_1[vvTight – Tight]/T	0.77	0.26	0.23	0.30	0.24	0.08	0.28
	Tight{Q,5} + c_1[vvTight – vTight]/T + c_2[vTight – Tight]/Q	c_1=2.45 c_2=0.59	0.08	0.05	0.05	0.12	-0.02	0.10
PNO-LCCSD(T)	Default {T,Q} + c_1[Tight – Default]/T	0.39	0.14	0.14	0.18	0.10	0.10	0.15
	Tight {T,Q} + c_1[Tight – Default]/T	0.30	0.17	0.19	0.25	0.10	0.12	0.19
	Tight {T,Q} + c_1[Tight-Default]/Q	0.66	0.19	0.21	0.27	0.11	0.13	0.21
DLPNO-CCSD(T_1)	NormalPNO{T,Q} + c_1[TightPNO – NormalPNO]/T	0.90	0.13	0.14	0.20	0.07	0.13	0.15
	NormalPNO{T,Q} + c_1[TightPNO/CPS{6,7} – NormalPNO]/T	0.93	0.14	0.11	0.16	0.14	0.13	0.15
	TightPNO{T,Q}+ c_1[VeryTightPNO-TightPNO]/T	1.02	0.25	0.23	0.31	0.20	0.10	0.27
	TightPNO{T,Q}/CPS + c_1[TightPNO/CPS - NormalPNO]/T	0.05	0.14	0.18	0.24	0.05	0.11	0.17
	(T_0)TightPNO/Q + c_1[(T_0)TightPNO/Q-(T_0)TightPNO/T] + c_2[(T_1)TightPNO/T-(T_0)TightPNO/T]	c_1=0.61 c_2=3.33	0.10	0.12	0.16	0.04	0.09	0.12
Coefficients (c_1) are optimized with respect to the revised ACONFL reference data.								
LNO-CCSD(T)	Normal{T,Q} + c_1[vTight – Normal]/T	-0.05	0.05	0.03	0.03	0.09	-0.01	0.07
	Normal{Q,5} + c_1[vTight – Normal]/T	0.45	0.13	0.07	0.10	0.18	0.11	0.16
	Tight{T,Q} + c_1[vTight – Tight]/T	-1.46	0.08	0.02	0.03	0.15	-0.06	0.12
	Tight{T,Q} + c_1[vvTight – Tight]/T	-1.42	0.08	0.03	0.03	0.15	-0.06	0.11
	Tight{Q,5} + c_1[vTight – Tight]/T	0.01	0.04	0.01	0.03	0.08	-0.04	0.06
	Tight{Q,5}+ c_1[vTight – Tight]/Q	0.05	0.04	0.01	0.03	0.08	-0.04	0.06
	Tight{Q,5} + c_1[vvTight – Tight]/T	0.01	0.04	0.01	0.03	0.08	-0.04	0.06
	vTight{T,Q} + c_1[vvTight – Tight]/T	-1.88	0.08	0.05	0.03	0.14	-0.06	0.11
	Tight {T,Q} + c_1[vTight – Tight]/T + c_2[vvTight – vTight]/T	c_1=-0.28 c_2=1.02	0.02	0.01	0.02	0.03	-0.02	0.03
PNO-LCCSD(T)	Default {T,Q} + c_1[Tight – Default]/T	1.63	0.05	0.01	0.03	0.10	0.05	0.08
	Tight {T,Q} + c_1[Tight – Default]/T	2.02	0.06	0.02	0.03	0.11	0.05	0.08
	Tight {T,Q} + c_1[Tight-Default]/Q	9.09	0.11	0.09	0.09	0.15	0.11	0.13
DLPNO-CCSD(T_1)	NormalPNO{T,Q} + c_1[TightPNO – NormalPNO]/T	0.41	0.05	0.01	0.04	0.07	-0.01	0.06
	NormalPNO{T,Q} + c_1[TightPNO/CPS{6,7} – NormalPNO]/T	0.48	0.04	0.02	0.04	0.04	0.00	0.05
	TightPNO{T,Q}+ c_1[VeryTightPNO-TightPNO]/T	-1.07	0.15	0.18	0.23	0.07	0.12	0.18
	TightPNO{T,Q}/CPS{6,7} + c_1[TightPNO/CPS{6,7} - NormalPNO]/T	-0.51	0.13	0.04	0.09	0.21	-0.05	0.17

DLPNO-CCSD(T_0)	NormalPNO{T,Q} + c_1[TightPNO - NormalPNO]/T	0.32	0.06	0.01	0.05	0.10	-0.02	0.08
	NormalPNO{T,Q} + c_1[TightPNO/CPS{6,7} - NormalPNO]/T	0.40	0.05	0.02	0.05	0.06	0.00	0.06
	TightPNO{T,Q} + c_1[VeryTightPNO-TightPNO]/T	-1.28	0.17	0.21	0.26	0.08	0.13	0.20
	TightPNO{T,Q}/CPS{6,7} + c_1[TightPNO/CPS{6,7} - NormalPNO]/T	-0.99	0.17	0.08	0.12	0.27	-0.05	0.23
	(T_0)TightPNO/Q + c_1[(T_0)TightPNO/Q-(T_0)TightPNO/T] + c_2[(T_1)TightPNO/T-(T_0)TightPNO/T]	c_1=1.25 c_2=16.81	0.03	0.03	0.03	0.04	0.00	0.04

[a]The expression CPS{X,Y} refers to the extrapolation of T_{CutPNO} to the complete PNO space limit using $T_{CutPNO}=10^{-X}$ and 10^{-Y}, where Y=X+1

Table S7: Performance of pure and composite LNO-CCSD(T), PNO-LCCSD(T), and DLPNO-CCSD(T_1) methods with respect to the revised reference conformer energies of *n*-hexadecane and *n*-icosane using HLC8. Heatmapping is from red (worst) via yellow to green (best).

Method Details	Threshold	Basis set	MAD (kcal/mol)				MSD	RMSD
			ACONFL	ACONF12	ACONF16	ACONF20	(kcal/mol)	(kcal/mol)
LNO-CCSD(T)	Normal	AVTZ	1.00	0.77	1.00	1.12	-0.11	1.10
		AVQZ	0.41	0.29	0.41	0.47	-0.03	0.45
		AV5Z	0.30	0.21	0.24	0.40	0.03	0.34
		AV{T,Q}Z	0.05	0.03	0.04	0.07	0.02	0.07
		AV{Q,5}Z	0.21	0.14	0.11	0.34	0.09	0.26
	Tight	AVTZ	0.85	0.60	0.82	1.02	-0.05	0.97
		AVQZ	0.26	0.16	0.24	0.34	0.00	0.31
		AV5Z	0.15	0.08	0.14	0.19	-0.01	0.17
		AV{T,Q}Z	0.12	0.13	0.14	0.10	0.04	0.13
		AV{Q,5}Z	0.03	0.01	0.04	0.04	-0.01	0.05
	vTight	AVTZ	0.77	0.51	0.71	0.97	0.00	0.90
		AVQZ	0.22	0.11	0.16	0.32	0.04	0.27
		AV5Z	0.10	0.04	0.07	0.17	0.03	0.13
		AV{T,Q}Z	0.15	0.15	0.20	0.11	0.06	0.16
		AV{Q,5}Z	0.03	0.05	0.04	0.02	0.03	0.04
	vvTight	AVTZ	0.77	0.50	0.71	0.96	0.00	0.89
PNO-LCCSD(T)	Default	AVTZ	0.06	0.04	0.04	0.09	0.02	0.07
		AVQZ	0.10	0.12	0.13	0.05	0.09	0.11
		AV5Z	—	0.10	0.16	—	—	—
		AV{T,Q}Z	0.15	0.18	0.21	0.09	0.14	0.17
		AV{Q,5}Z	—	0.08	0.18	—	—	—
	Tight	AVTZ	0.12	0.07	0.12	0.14	-0.02	0.13
		AVQZ	0.09	0.11	0.11	0.07	0.09	0.10
		AV5Z	—	0.10	—	—	—	—
		AV{T,Q}Z	0.18	0.23	0.26	0.08	0.16	0.20
		AV{Q,5}Z	—	0.10	—	—	—	—
DLPNO-CCSD(T_0)	NormalPNO	AVTZ	0.77	0.68	0.90	0.72	-0.22	0.84
		AVQZ	0.33	0.32	0.41	0.26	-0.14	0.36
		AV5Z	0.24	0.28	0.35	0.14	-0.16	0.28
		AV{T,Q}	0.09	0.09	0.09	0.10	-0.09	0.10
		AV{Q,5}	0.20	0.23	0.29	0.11	-0.19	0.23
	TightPNO $T_{CutPNO}=10^{-6}$	AVTZ	0.56	0.30	0.45	0.78	0.07	0.67
		AVQZ	0.13	0.10	0.11	0.17	-0.01	0.15
		AV5Z	—	0.16	—	—	—	—
		AV{T,Q}	0.17	0.05	0.12	0.27	-0.06	0.22
		AV{Q,5}	—	0.42	—	—	—	—
	TightPNO $T_{CutPNO}=10^{-7}$	AVTZ	0.67	0.38	0.57	0.91	0.06	0.79
		AVQZ	0.18	0.04	0.08	0.35	0.11	0.26
		AV5Z	—	0.05	—	—	—	—
		AV{T,Q}	0.18	0.23	0.28	0.06	0.14	0.22
		AV{Q,5}	—	0.11	—	—	—	—
	TightPNO	AVTZ	0.72	0.42	0.62	0.98	0.05	0.86

	T_{CutPNO}=$10^{-\{6,7\}}$ or CPS{6,7}[a]	AVQZ	0.23	0.06	0.10	0.44	0.17	0.33
		AV5Z	—	0.02	—	—	—	—
		AV{T,Q}	0.26	0.33	0.37	0.14	0.25	0.29
	VeryTightPNO	AV{Q,5}	—	0.05	—	—	—	—
		AVTZ	0.61	0.36	0.53	0.81	0.05	0.71
		AVQZ	—	0.01	—	—	—	—
		AV{T,Q}	—	0.25	—	—	—	—
DLPNO-CCSD(T_1)	NormalPNO	AVTZ	0.79	0.70	0.93	0.72	-0.23	0.86
		AVQZ	0.34	0.35	0.44	0.26	-0.16	0.38
		AV5Z	0.26	0.30	0.38	0.14	-0.18	0.30
		AV{T,Q}Z	0.11	0.11	0.12	0.11	-0.10	0.12
		AV{Q,5}Z	0.22	0.25	0.32	0.12	-0.20	0.25
	TightPNO T_{CutPNO}=10^{-6}	AVTZ	0.58	0.32	0.49	0.81	0.06	0.69
		AVQZ	0.15	0.05	0.07	0.27	0.12	0.20
		AV5Z	—	0.13	—	—	—	—
		AV{T,Q}Z	0.24	0.29	0.34	0.12	0.16	0.27
		AV{Q,5}Z	—	0.22	—	—	—	—
	TightPNO T_{CutPNO}=10^{-7}	AVTZ	0.70	0.41	0.60	0.94	0.05	0.82
		AVQZ	0.20	0.05	0.09	0.37	0.10	0.28
		AV5Z	—	0.03	—	—	—	—
		AV{T,Q}Z	0.16	0.20	0.25	0.06	0.13	0.19
		AV{Q,5}Z	—	0.08	—	—	—	—
	TightPNO T_{CutPNO}=$10^{-\{6,7\}}$ or CPS{6,7}[a]	AVTZ	0.76	0.45	0.66	1.01	0.05	0.89
		AVQZ	0.25	0.08	0.14	0.43	0.09	0.33
		AV5Z	—	0.04	—	—	—	—
		AV{T,Q}Z	0.14	0.16	0.20	0.07	0.12	0.16
		AV{Q,5}Z	—	0.02	—	—	—	—
	VeryTightPNO	AVTZ	0.64	0.39	0.56	0.84	0.04	0.75
		AVQZ	—	0.02	—	—	—	—
		AV{T,Q}Z	—	0.23	—	—	—	—

[a]The expression CPS{X,Y} refers to the extrapolation of T_{CutPNO} to the complete PNO space limit using T_{CutPNO}=10^{-X} and 10^{-Y}, where Y=X+1

Table S8: Performance of standard and composite PNO-LCCSD(T) methods with respect to the revised reference conformer energies of longer n-alkanes. The default ″REXT″ setting for the "Tight" and "Default" domains (7 and 5 bohr, respectively) were used throughout. Heatmapping is from red (worst) via yellow to green (best).

Threshold	Basis set	c_1	MAD (kcal/mol)				MSD (kcal/mol)	RMSD (kcal/mol)
			ACONFL	ACONF12	ACONF16	ACONF20		
Default	AVTZ		0.05	0.04	0.06	0.05	0.02	0.06
	AVQZ		0.10	0.11	0.15	0.05	0.07	0.12
	AV5Z			0.10				
	AV{T,Q}Z		0.16	0.17	0.22	0.10	0.11	0.18
	AV{Q,5}Z			0.09				
Tight	AVTZ		0.42	0.37	0.46	0.41	-0.10	0.46
	AVQZ		0.05	0.02	0.02	0.09	0.03	0.07
	AV5Z			0.05				
	AV{T,Q}Z		0.21	0.22	0.29	0.15	0.12	0.23
	AV{Q,5}Z			0.11				
Composite methods[a]	Default {T,Q} + c_1[Tight – Default]/T	0.39	0.06	0.01	0.03	0.12	0.06	0.08
	Tight {T,Q} + c_1[Tight – Default]/T	0.30	0.09	0.10	0.13	0.06	0.08	0.11
	Tight {T,Q} + c_1[Tight-Default]/Q	0.66	0.12	0.14	0.18	0.07	0.09	0.14
Composite methods[b]	Default {T,Q} + c_1[Tight – Default]/T	0.40	0.06	0.01	0.03	0.12	0.06	0.09
	Tight {T,Q} + c_1[Tight – Default]/T	0.53	0.06	0.01	0.03	0.11	0.05	0.07
	Tight {T,Q} + c_1[Tight-Default]/Q	1.53	0.05	0.03	0.04	0.08	0.05	0.06

[a]Coefficients are taken from the “raw” category of Table 4 in Ref.[2]

[b]Coefficients are reoptimized relative to the revised ACONFL reference data.

Table S9: Performance of explicitly correlated PNO-LCCSD(T)-F12b with respect to the revised reference conformer energies of longer n-alkanes. The default "REXT" setting for the "Tight" and "Default" domains (7 and 5 a.u., respectively) were used throughout. Heatmapping is from red (worst) via yellow to green (best).

Methods	Threshold	Basis set	MAD (kcal/mol)				MSD (kcal/mol)	RMSD (kcal/mol)
			ACONFL	ACONF12	ACONF16	ACONF20		
PNO-LCCSD(T)-F12b	Default	VDZ-F12	0.17	0.08	0.12	0.26	-0.05	0.20
		VTZ-F12	0.16	0.14	0.19	0.15	0.04	0.18
		VQZ-F12		0.10				
	Tight	VDZ-F12	0.16	0.13	0.16	0.17	0.01	0.17
		VTZ-F12	0.13	0.12	0.16	0.12	0.04	0.14
PNO-LCCSD(Ts)-F12b[a]	Default	VDZ-F12	0.10	0.02	0.04	0.18	-0.06	0.13
		VTZ-F12	0.13	0.12	0.15	0.12	0.04	0.14
		VQZ-F12		0.09				
	Tight	VDZ-F12	0.08	0.06	0.07	0.09	0.00	0.08
		VTZ-F12	0.10	0.09	0.12	0.08	0.03	0.10

[a]Following Ref.[3], the (T) terms of PNO-LCCSD(T)-F12b and DLPNO-CCSD(T_1)-F12 were scaled by 1.1413, 1.0527, and 1.0232 for VDZ-F12, VTZ-F12, and VQZ-F12, respectively.

The complete author list of Q-CHEM 6

Evgeny Epifanovsky, Andrew T. B. Gilbert, Xintian Feng, Joonho Lee, Yuezhi Mao, Narbe Mardirossian, Pavel Pokhilko, Alec F. White, Marc P. Coons, Adrian L. Dempwolff, Zhengting Gan, Diptarka Hait, Paul R. Horn, Leif D. Jacobson, Ilya Kaliman, Jörg Kussmann, Adrian W. Lange, Ka Un Lao, Daniel S. Levine, Jie Liu, Simon C. McKenzie, Adrian F. Morrison, Kaushik D. Nanda, Felix Plasser, Dirk R. Rehn, Marta L. Vidal, Zhi-Qiang You, Ying Zhu, Bushra Alam, Benjamin J. Albrecht, Abdulrahman Aldossary, Ethan Alguire, Josefine H. Andersen, Vishikh Athavale, Dennis Barton, Khadiza Begam, Andrew Behn, Nicole Bellonzi, Yves A. Bernard, Eric J. Berquist, Hugh G. A. Burton, Abel Carreras, Kevin Carter-Fenk, Romit Chakraborty, Alan D. Chien, Kristina D. Closser, Vale Cofer-Shabica, Saswata Dasgupta, Marc de Wergifosse, Jia Deng, Michael Diedenhofen, Hainam Do, Sebastian Ehlert, Po-Tung Fang, Shervin Fatehi, Qingguo Feng, Triet Friedhoff, James Gayvert, Qinghui Ge, Gergely Gidofalvi, Matthew Goldey, Joe Gomes, Cristina E. González-Espinoza, Sahil Gulania, Anastasia O. Gunina, Magnus W. D. Hanson-Heine, Phillip H. P. Harbach, Andreas Hauser, Michael F. Herbst, Mario Hernández Vera, Manuel Hodecker, Zachary C. Holden, Shannon Houck, Xunkun Huang, Kerwin Hui, Bang C. Huynh, Maxim Ivanov, Ádám Jász, Hyunjun Ji, Hanjie Jiang, Benjamin Kaduk, Sven Kähler, Kirill Khistyaev, Jaehoon Kim, Gergely Kis, Phil Klunzinger, Zsuzsanna Koczor-Benda, Joong Hoon Koh, Dimitri Kosenkov, Laura Koulias, Tim Kowalczyk, Caroline M. Krauter, Karl Kue, Alexander Kunitsa, Thomas Kus, István Ladjánszki, Arie Landau, Keith V. Lawler, Daniel Lefrancois, Susi Lehtola, Run R. Li, Yi-Pei Li, Jiashu Liang, Marcus Liebenthal, Hung-Hsuan Lin, You-Sheng Lin, Fenglai Liu, Kuan-Yu Liu, Matthias Loipersberger, Arne Luenser, Aaditya Manjanath, Prashant Manohar, Erum Mansoor, Sam F. Manzer, Shan-Ping Mao, Aleksandr V. Marenich, Thomas Markovich, Stephen Mason, Simon A. Maurer, Peter F. McLaughlin, Maximilian F. S. J. Menger, Jan-Michael Mewes, Stefanie A. Mewes, Pierpaolo Morgante, J. Wayne Mullinax, Katherine J. Oosterbaan, Garrette Paran, Alexander C. Paul, Suranjan K. Paul, Fabijan Pavošević, Zheng Pei, Stefan Prager, Emil I. Proynov, Ádám Rák, Eloy Ramos-Cordoba, Bhaskar Rana, Alan E. Rask, Adam Rettig, Ryan M. Richard, Fazle Rob, Elliot Rossomme, Tarek Scheele, Maximilian Scheurer, Matthias Schneider, Nickolai Sergueev, Shaama M. Sharada, Wojciech Skomorowski, David W. Small, Christopher J. Stein, Yu-Chuan Su, Eric J. Sundstrom, Zhen Tao, Jonathan Thirman, Gábor J. Tornai, Takashi Tsuchimochi, Norm M. Tubman, Srimukh Prasad Veccham, Oleg Vydrov, Jan Wenzel, Jon Witte, Atsushi Yamada, Kun Yao, Sina Yeganeh, Shane R. Yost, Alexander Zech, Igor Ying Zhang, Xing Zhang, Yu Zhang, Dmitry Zuev, Alán Aspuru-Guzik, Alexis T. Bell, Nicholas A. Besley, Ksenia B. Bravaya, Bernard R. Brooks, David Casanova, Jeng-Da Chai, Sonia Coriani, Christopher J. Cramer, György Cserey, A. Eugene DePrince III, Robert A. DiStasio Jr., Andreas Dreuw, Barry D. Dunietz, Thomas R. Furlani, William A. Goddard III, Sharon Hammes-Schiffer, Teresa Head-Gordon, Warren J. Hehre, Chao-Ping Hsu, Thomas-C. Jagau, Yousung Jung, Andreas Klamt, Jing Kong, Daniel S. Lambrecht, WanZhen Liang, Nicholas J. Mayhall, C. William McCurdy, Jeffrey B. Neaton, Christian Ochsenfeld, John A. Parkhill, Roberto Peverati, Vitaly A. Rassolov, Yihan Shao, Lyudmila V. Slipchenko, Tim Stauch, Ryan P. Steele, Joseph E. Subotnik, Alex J. W. Thom, Alexandre Tkatchenko, Donald G. Truhlar, Troy Van Voorhis, Tomasz A. Wesolowski, K. Birgitta Whaley, H. Lee Woodcock III, Paul M. Zimmerman, Shirin Faraji, Peter M. W. Gill, Martin Head-Gordon, John M. Herbert, and Anna I. Krylov. *Software for the frontiers of quantum chemistry: An overview of developments in the Q-Chem 5 package.* [*J. Chem. Phys.*. 155, 084801 (2021)]

See also: https://www.q-chem.com/

References:

1 S. Ehlert, S. Grimme and A. Hansen, Conformational Energy Benchmark for Longer n - Alkane Chains, *J. Phys. Chem. A*, 2022, **126**, 3521–3535.

2 G. Santra, E. Semidalas, N. Mehta, A. Karton and J. M. L. Martin, S66x8 noncovalent interactions revisited: new benchmark and performance of composite localized coupled-cluster methods, *Phys. Chem. Chem. Phys.*, 2022, **24**, 25555–25570.

3 K. A. Peterson, M. K. Kesharwani and J. M. L. Martin, The `cc-pV5Z-F12 basis set: Reaching the basis set limit in explicitly correlated calculations, *Mol. Phys.*, 2015, **113**, 1551–1558.
